# Preliminary Investigation of Microdosimetric Track Structure Physics Models in Geant4-DNA and RITRACKS

**DOI:** 10.1155/2015/968429

**Published:** 2015-06-01

**Authors:** Michael Douglass, Scott Penfold, Eva Bezak

**Affiliations:** Royal Adelaide Hospital, North Terrace, Adelaide, SA 5000, Australia

## Abstract

The major differences between the physics models in Geant4-DNA and RITRACKS Monte Carlo packages are investigated. Proton and electron ionisation interactions and electron excitation interactions in water are investigated in the current work. While these packages use similar semiempirical physics models for inelastic cross-sections, the implementation of these models is demonstrated to be significantly different. This is demonstrated in a simple Monte Carlo simulation designed to identify differences in interaction cross-sections.

## 1. Introduction

Condensed history transport models for charged particle transport have been used extensively since it was first implemented in 1963 [1]. Decades of research and experience have given us confidence that this type of physics model can accurately predict macroscopic quantities such as dose deposition.

Research into the microscopic scale effects of radiation on cellular behaviour is becoming increasingly popular. Condensed history physics models are unsuitable for this type of investigation as the crucial microscopic track structure responsible for cellular and chromosomal damage is not simulated in detail. Simulating particle interactions on molecular scales requires highly accurate experimental data and equally robust semiempirical and theoretical models.

**Figure 1:**
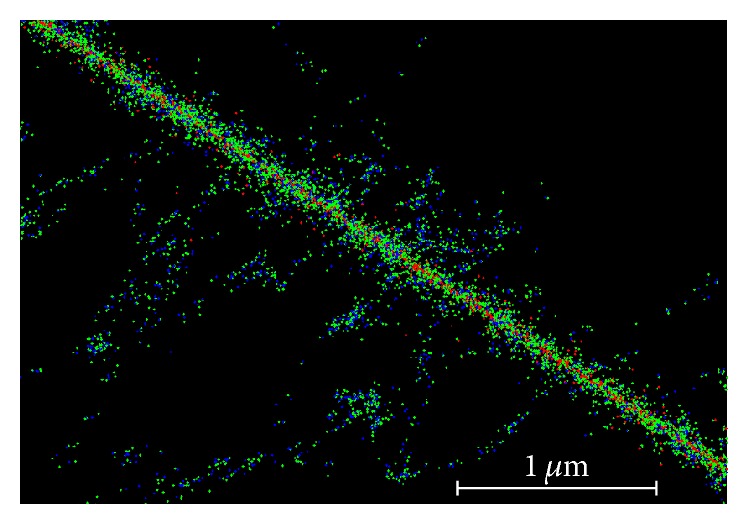
The track structure of a single proton in water. Simulated using RITRACKS. Red: proton ionisation, green: electron ionisation, and blue: hydroxyl radicals.

Geant4-DNA and other Monte Carlo packages including RITRACKS include physics models which enable the path of charged particles and all successive interactions to be simulated and tracked on nanoscopic scales (Figure 1). The package included in the Geant4 distribution is called Geant4-DNA [2]. Geant4-DNA has the ability to simulate the passage of charged particles in a liquid water medium and models each and every particle down to energies of the order of 10 eV and 100 eV for electrons and protons/hydrogen, respectively.

Geant4-DNA is packaged as part of the Geant4 [3] distribution. Geant4 has been developed and is maintained by an international collaboration of scientists and engineers. It has applications in areas such as high energy physics, astrophysics, and medical physics. Geant4-DNA includes physics models to simulate the track structure of electrons, protons, and several heavy ions.

RITRACKS [4] is a Monte Carlo (MC) particle tracking software designed to simulate the interactions of cosmic radiation in a space environment. It was developed by Ianik Plante of the NASA Johnson Space Centre in 2011. It is capable of simulating all the interactions of cosmic radiation and electrons in liquid water.

Geant4-DNA and RITRACKS are similar in terms of their ability to simulate radiation track structure. However, each package uses different physics models (or different implementation of the same model). The purpose of this study was to compare the physics models used in each simulation (Geant4 v9.6 p01 and RITRACKS v3.1) and the simulation results which they produce.

In the current work, a review was performed to investigate the implementation of the microdosimetry models in two MC packages. The cross-sections implemented by the two MC packages were compared against experimentally obtained values. A simple MC simulation was then performed using both packages to compare their predictions. This investigation was performed in order to estimate the energy range for protons and electrons in which we can confidently assume that simulation predictions are accurate.

## 2. Overview of Microdosimetry Physics Models

### 2.1. Electron Ionisation

Ionisation cross-sections for electrons in water are calculated in RITRACKS using two distinct models covering two different energy ranges. The Rudd model [5] is used for electron energies between 1 eV and 50 keV and Seltzer's model [6, 7] for energies over 50 keV.

Rudd [5] derived a semiempirical equation for the differential cross-section for electron ionisation based on the Mott equation (parameters shown in Table 1). The Pauli principle states that the secondary electrons in ionisation interactions are indistinguishable from the primary electrons which leads to

(1)  dσionidω=dσioni1dω+dσioni2dω,dσioni1dω =SiIiF1t11+ω3+1t−ω3−11+ω3/2t−ω3/2,dσioni2dω =SiIiF2t11+ω2+1t−ω2−11+ωt−ω,

where *t* = *T*/*I*_*i*_ and *ω* = *W*/*I*_*i*_, *T* is the energy of the primary electron, *W* is the energy of the ejected electron, *I*_*i*_ is the binding energy of the electron in the *i*th molecular orbital, *S*_*i*_ = 4*πa*_0_^2^ *N*_*i*_(*ℜ*/*I*_*i*_)^2^, *a*_0_ is the Bohr radius, *ℜ* is the Rydberg constant, and *N*_*i*_ is the number of electrons in the orbital [8]:

(2) F1t=A1ln⁡tt+B1,F2t=A2ln⁡tt+B2,

where *A*_1_, *A*_2_, *B*_1_, and *B*_2_ are constants determined through fitting of experimental data.

Above 50 keV, RITRACKS uses Seltzer's formula (parameters shown in Table 2) for the differential cross-section of electron ionisation [6, 7]. This formula is used to calculate the cross-section for the *i*th orbital of the water molecule for a secondary electron with energy *W* and an incident electron with kinetic energy *T*. It is the sum of the close collision and distant collision contributions:

(3) dσionidW=dσcidW+dσdidW,dσcidW=1E2+1T−W2+1T2ττ+12 − 2τ+1τ+121ET−W+Gi×2πre2mc2Niβ2TT+Bi+Ui,Gi=8Ui3π1E3+1T−W3tan−1y+yy−1y+12,dσdidW=NiIEσPEiE,

where *σ*_PE_^*i*^(*E*) is the photoelectric cross-section for the *i*th molecular orbital of water for a photon with incident energy *E* = *W* + *B*_*i*_, *τ* is the kinetic energy of the electron in units of electron rest mass, *U*_*i*_ is the mean kinetic energy of the target electron in the orbital, *r*_*e*_ = 2.817 × 10^−15^ m is the electron radius, *β* = *v*/*c*, *B*_*i*_ is the binding energy, and *y* = *W*/*U*_*i*_.

### 2.2. Electron Excitation

The electron excitation differential cross-section in water is calculated in RITRACKS using two different models. The two models cover the energy range from <100 eV and >100 eV, respectively. The cross-sections in the higher energy range are calculated using the model of Kutcher and Green [9]:

(4) $$\begin{array}{c} \frac{d\sigma_{ex}^{i}}{dW}=\rho \left(W\right)Wf_{i}\left(W\right)ln\left[\frac{4T}{Q_{min}}\right], \end{array}$$

where *T* is the energy of the incident electron, *W* is the energy lost by the electron through excitation of the water molecule, *f*_*i*_(*W*) are Gaussian functions representing excitation levels, *ρ*(*W*) is the differential cross-section for charged particles on free electrons at rest, and *Q*_min_ is the minimum energy that is transferred in an interaction [8]:

(5) Qmin=2T1−12WT−1−WT,ρW=e48πε02mυ2W2=4πa02TRW2,

where *a*_0_ is the Bohr radius, *ε*_0_ is the vacuum permittivity, and *ℜ* is the Rydberg constant.

In the energy range where the electron energy is close to the excitation energy of the molecule, the above model is not valid. For electrons of energy less than 100 eV, Kaplan and Sukhonosov [10] and Cobut [11] developed the following model for calculating the electron excitation differential cross-section:

(6) $$\begin{array}{c} \frac{d\sigma_{ex}^{i}}{dW}=\rho \left(W\right)Wf_{i}\left(W\right)ln\left[\frac{\alpha \left(t\right)T}{W}\right], \end{array}$$

where *α*(*t*) = 4 − 3exp[−(*W* − *W*_0,*i*_)/*α*_*i*_], *α*_*i*_ = 4*E*_min_/ln(2), *E*_min_ = 7.34 eV is the minimum energy transfer by excitation, and *W*_0,*i*_ is a parameter (units of energy) which is used to link the excitation cross-sections at low and high energies [8].

### 2.3. Proton Ionisation

The ionisation differential cross-section for protons and heavy ions in water is calculated in RITRACKS using a semiempirical equation. The equation was proposed by Rudd [5] for the differential cross-section of each molecular orbital of liquid water by protons (7).

A relativistic correction has been applied to the semiempirical cross-sections for proton ionisation and excitation in water molecules to extend the cross-sections to 10 GeV/amu. The nonrelativistic formula is [12]

(7) $$\begin{array}{c} \frac{d\sigma_{ion}^{i}}{d\omega }=\frac{S_{i}}{I_{i}}\frac{F_{1}\left(\upsilon \right)+\omega F_{2}\left(\upsilon \right)}{I_{i}{\left(1+w\right)}^{3}\left(1+\exp\left(\alpha \left(\omega -\omega_{c_{i}}\right)/\upsilon \right)\right)}, \end{array}$$

where *i* is the index of the orbital, *I*_*i*_ is the binding energy of the electron in the target, *ω* = *W*/*I*_*i*_, *W* is the energy of the secondary electron, and *E*_*p*_ is the energy of the incident proton. *F*_1_ and *F*_2_ consist of a series of values fitted from experimental data. The molecular orbits considered in the model are the 1*b*_1_, 3*a*_1_, 1*b*_2_, 2*a*_1_, and 1*a*_1_. *T* = *E*_*p*_(*m*/*M*_*p*_) is the kinetic energy of an electron of mass *m* which would have the same velocity as a proton of mass *M*_*p*_ and $\upsilon =\sqrt{T/I_{i}}$ is a scaled velocity of the incident particle.

This model is valid only in the classical energy range. In [12], the classical model was extended to the relativistic energy range by making the substitution

(8) $$\begin{array}{c} \upsilon^{2}=\frac{mc^{2}}{2I_{i}}\left[1-\frac{1}{{\left(1+T/mc^{2}\right)}^{2}}\right]. \end{array}$$

For track structure simulations of protons and heavy ions, the first order plane wave Born approximation is used. To obtain the interaction cross-sections for heavy ions, the cross-section for protons (of the same velocity *υ* as the heavy ion) is scaled by the square of the charge *Z* of the ion [12, 13]:

(9) $$\begin{array}{c} \frac{d\sigma_{ion}\left(\upsilon \right)}{dW}=Z^{2}\frac{d\sigma_{proton}\left(\upsilon \right)}{dW}. \end{array}$$

Equation (10) can be used to calculate the kinetic energy of an ion which has the same velocity as a proton with kinetic energy *τ*. This formulation is true for both relativistic and nonrelativistic ions:

(10) $$\begin{array}{c} E_{ion}=\frac{M}{M_{p}}\tau . \end{array}$$

In some cases, heavy ions at low energies may have a reduced effective charge due to the attachment of electrons. In order to accurately calculate the cross-sections for both ionisation and excitation in a water medium, the “reduced” charge must be calculated. Booth and Grant [14] derived an equation for the effective charge of an ion:

(11) Z∗Z=1−exp−1.316x+0.112x2−0.0650x3,iiiiiiiiiiiiiiiiiiiiiiiiiiiiiiiiiiiiiiwhere  x=100βZ−2/3.

This correction term becomes significant at approximately 0.2 MeV for protons [12].

## 3. Geant4-DNA Physics Models

Geant4-DNA implements similar models to RITRACKS for calculating the differential cross-sections of protons, electrons, alpha particles, and heavy ions. All possible physical interactions are taken into account such as ionisation, excitation, charge transfer, and elastic scattering [15].

Geant4-DNA calculates inelastic cross-sections for protons using the Rudd ionisation model for low energies and plane wave first Born approximation [16, 17] for energies above 500 keV. While the proton cross-sections are available for energies between 100 eV and 100 MeV, the cross-sections have not been verified experimentally below 1 keV.

The Born double differential inverse mean free path is given by [15]

(12) d2σT,E,qdE·dq =1πα0TqIm−1εE,qθq−q−E,τ  ×θq+E,τ−qθτ−E,

where *α*_0_ is the Bohr radius, *τ* is the particle kinetic energy, *T* = (*m*/*M*)*τ* is the kinetic energy of an electron travelling with the same velocity of the considered particle, *m* is the electron mass, *M* is the particle mass, *θ* is the heavy side step function, and *ε* = *ε*_1_ + *iε*_2_ is the complex dielectric response function of the material in which the particle is propagating and [15]

(13) $$\begin{array}{c} q_{\pm }=\sqrt{2M}\left(\sqrt{\tau }\pm \sqrt{\tau -E}\right) \end{array}$$

is the momentum transfer.

When the speed of the incident particle approaches the speed of the electrons orbiting the water molecule in the medium (<1 keV for electrons and <300 keV for protons), the first Born approximation is no longer valid. Proton ionisation cross-sections were corrected using the Rudd ionisation model [15]. Electron cross-sections were corrected using a Coulomb field correction proposed in ICRU 37 [18].

Ionisation cross-sections for heavy ions (including Li, Be, B, C, N, O, Si, and Fe) are calculated in Geant4-DNA using the extended Rudd ionisation model. The energy range covered by this model is 0.5 MeV/amu–1 GeV/amu.

Ionisation cross-sections for protons, hydrogen, alpha, and charged ion states in water are calculated in Geant4-DNA using the Rudd ionisation model (and Born model for protons above 500 keV). Proton, hydrogen, and alpha excitation cross-sections are calculated using the Miller and Green model [19].

Electron interactions can be simulated by default using cross-sections calculated using the Born ionisation model from 7.4 eV to relativistic energies of 1 MeV. Electrons with energy below 8 eV do not have sufficient energy to ionise a water molecule. However, these electrons still have sufficient energy to undergo vibrational and rotational excitations of the water medium until the electron finally reaches thermalisation. At the current time, there are no theoretical models which accurately describe this process. For Geant4-DNA, experimental cross-sections for ice targets published by [20] are utilised for electron excitation by performing a phase-scaling correction. This correction enabled the cross-sections to be used for a liquid water medium [15].

Alpha particle (and other heavy ions) cross-sections are calculated in Geant4-DNA using the same method implemented in RITRACKS (scaling proton cross-sections by the squared charge of the ion). Similarly, cross-sections for ions with bound electrons (at low energies) are corrected using the same method as RITRACKS (*Z*_*∗*_ method).

In Geant4-DNA, the inelastic (ionisation) cross-sections are calculated using five ionisation and five excitation states for water. Both RITRACKS and Geant4-DNA consider particle interactions in a water medium only due to limited amounts of experimental data for verification and semiempirical model development. However, water is considered to be the primary component of cells and therefore both these models are thought to replicate the physical interactions in cells with reasonable accuracy.

## 4. Methods

In this section, the published cross-sections for Geant4-DNA are compared with published experimental data and RITRACKS models in order to estimate the energy range for protons and electrons in which we can confidently assume that the simulation predictions are accurate.

The physical interactions of significant interest in the field of heavy ion radiobiology are proton ionisation, electron ionisation, and electron excitation processes. These processes dominate the energy loss mechanisms of protons and contribute most significantly to the biological damage of cells.

A review was performed to obtain several independent experimental and analytical data sets for the interaction cross-sections of protons and electrons. This data was compared with the cross-sections published for Geant4-DNA and RITRACKS.

**Figure 3:**
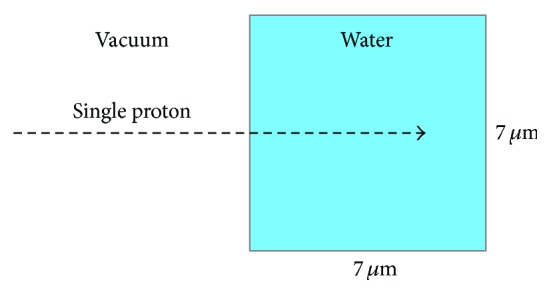
The simulation geometry used to compare particle transport in Geant4-DNA and RITRACKS.

The ionisation tracks of protons and electrons in water using Geant4-DNA and RITRACKS were then compared. A small cubic water volume with dimensions 7 *μ*m × 7 *μ*m × 7 *μ*m was used as a target for radiation interactions in both simulations. A single proton was fired into the water volume and the total number of primary and secondary ionisation events in the water volume was recorded (Figure 3). The simulation was run multiple times and the values were averaged in order to obtain the statistical uncertainty. The energy of the incident proton was then varied between 10^−2^ and 10^2^ MeV and the total number of ionisation events was recorded. This simple simulation utilises all the proton and electron cross-sections and models characterised earlier.

## 5. Results

**Figure 4:**
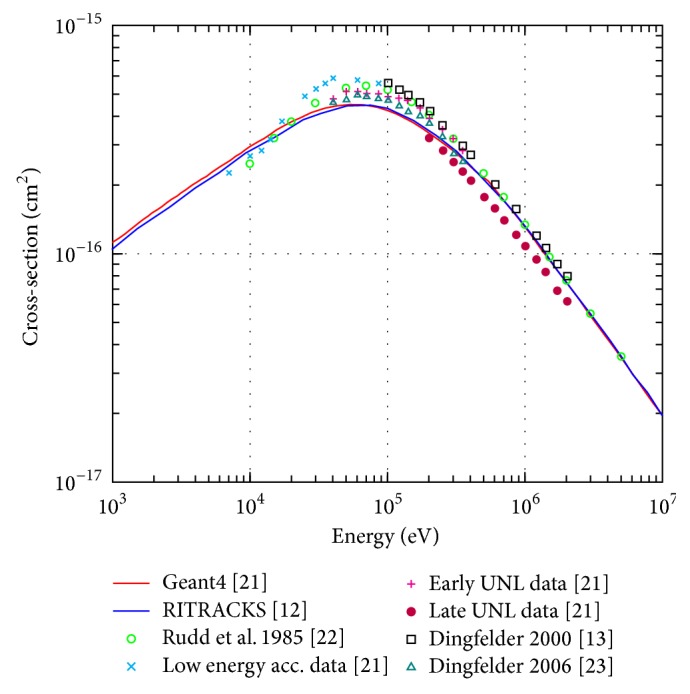
Total ionisation cross-section for protons in liquid water in the energy range of 1 keV to 10 MeV.

In Figure 4, the total interaction cross section for proton ionisation interactions is shown in the energy range of 1 keV to 10 MeV. The calculated cross sections for Geant4-DNA [21] and RITRACKS [8] are compared with experimental data from [13, 21–23]. As expected, there is excellent agreement between Geant4-DNA and RITRACKS predictions as each package uses similar implementations of the same model (the Rudd ionisation model) (Tables 3 and 4). Although Geant4-DNA utilises the Born ionisation model for proton energies above 500 keV while RITRACKS uses the Rudd model for all energies, there is still excellent agreement.

**Figure 5:**
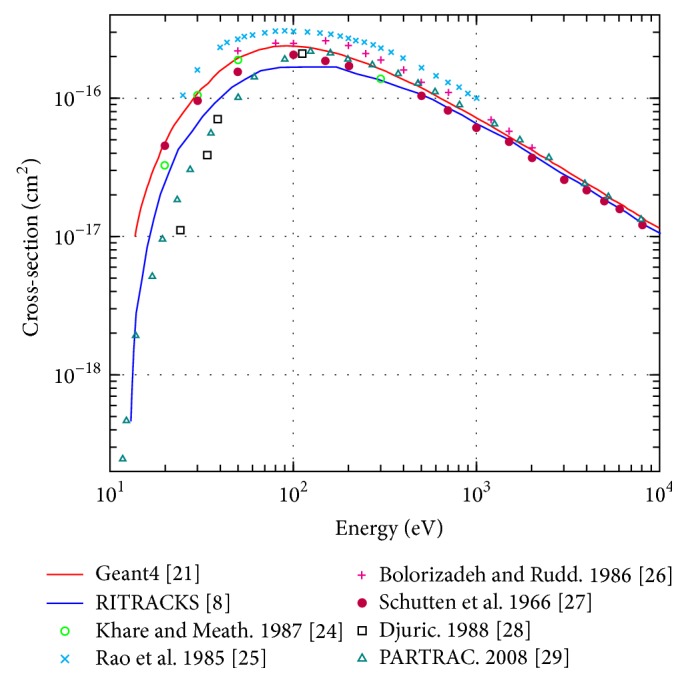
Total ionisation cross-section for electrons in liquid water in the energy range of 10 eV to 10 keV.

Figure 5 compares the total ionisation cross section for electrons in liquid water between energies of 10 eV and 10 keV using Geant4-DNA and RITRACKS and experimental data from [24–29]. There is still excellent agreement between the two simulations above energies of 1 keV. However, the models used to predict electron interactions differ between the two simulations resulting in a significant difference in cross sections at very low energies (factor of 200 difference at 13 eV). It appears that Geant4-DNA predicted data is generally in better agreement with experimental data over the entire applicable energy range.

**Figure 6:**
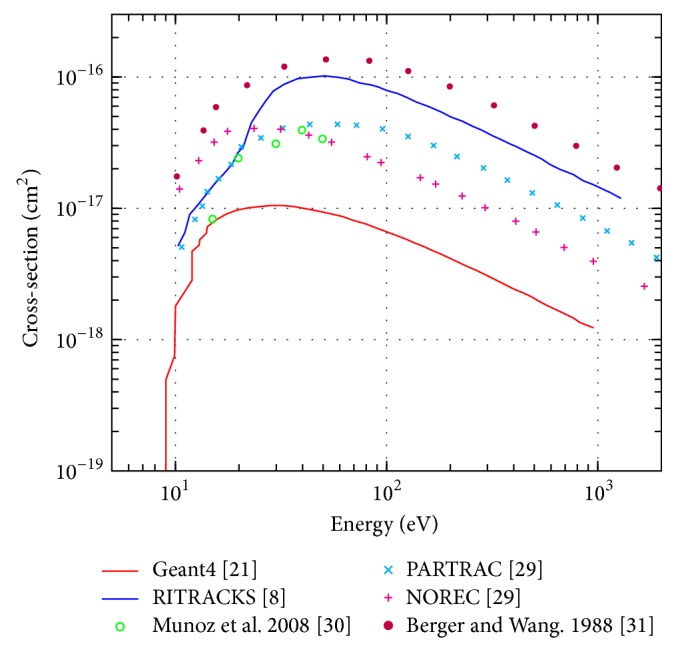
Total excitation cross-section for electrons in liquid water in the energy range of 1 eV to 1 keV.

In the case of electronic excitation processes, Geant4-DNA and RITRACKS again utilise different models to predict cross sections. The two different simulations predict significantly different interaction cross sections for all energies (an order of magnitude difference). In addition to the experimental data [29–31] in Figure 6, the predicted data from two additional MC codes (PARTRAC and NOREC) are plotted on the same axes. All four MC packages give significantly different values for electronic excitation cross sections. The accuracy of these cross sections cannot be validated with certainty at this time due to lack of experimental data.

**Figure 7:**
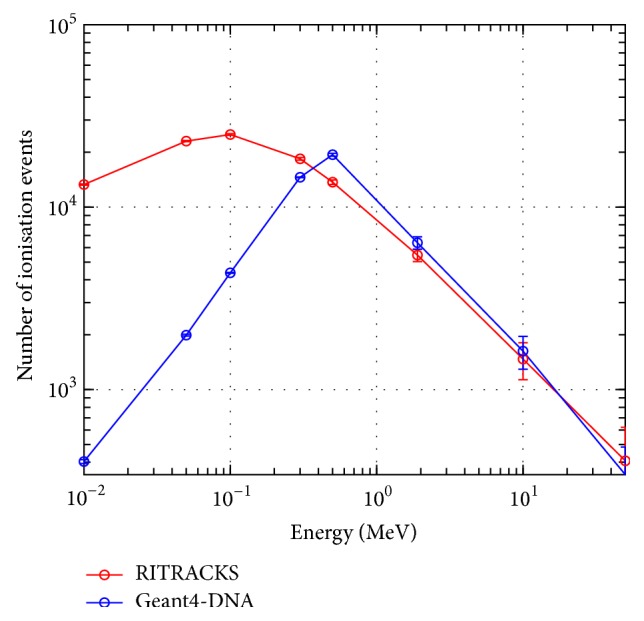
Total number of ionisation events recorded in a 7 *μ*m × 7 *μ*m × 7 *μ*m water box from the passage of a single proton and secondary electrons in liquid water using Geant4-DNA and RITRACKS.

Figure 7 shows the total number of ionisation events produced by the passage of a single proton versus the energy of the incident proton. There is reasonable agreement between RITRACKS and Geant4 (v9.6 patch 01) for proton energies above 0.25 MeV. Below 0.25 MeV, there is a large discrepancy between the total number of ionisation events recorded using each simulation code.

## 6. Discussion and Conclusion

A preliminary investigation of the physics models for proton and electron ionisation interactions used in RITRACKS and Geant4-DNA has been performed. Proton and heavy ion interaction cross-sections are implemented with similar semiempirical models in both Geant4-DNA and RITRACKS. This is reflected by the agreement in the data predicted using Geant4-DNA and RITRACKS.

There is excellent agreement between Geant4-DNA, RITRACKS, and experimental data for proton ionisation cross-sections in the energy range of 10 keV to 5 MeV. Geant4-DNA and RITRACKS use the same model to predict interaction cross-sections in this energy range (Geant4-DNA utilises the Born bonisation model above 0.5 MeV) resulting in very similar cross-sections. Electron ionisation cross-sections are calculated with different models resulting in large discrepancies which increase below energies of 200–300 eV. At energies above 300 eV, there is good agreement between both models and experimental data. Electron excitation cross-sections are calculated in each MC package using different models. There is an order of magnitude difference between the predicted cross-sections at energies below 1 keV. There is also insufficient experimental data to verify the accuracy of either model at this time.

A simple simulation was performed with both MC models using the same geometry. The number of ionisation events in a small water volume produced by a single proton of different energies was recorded. RITRACKS and Geant4-DNA produce similar results for proton energies above 500 keV. However, below 500 keV, there is more than one order of magnitude difference in the total number of ionisation events. It is unclear from the comparison of cross-sections between the two MC codes where this discrepancy is derived from, although it should be noted that the current version of RITRACKS (v3.2) has been modified (since v3.1) to limit incident protons to energies above 0.1 MeV.

Both RITRACKS and Geant4-DNA use semiempirical models to predict the interaction cross-sections for ionisation and excitation events of protons and electrons. The accuracy of the predictions made by these models depends on accurate experimental measurements. Below 1 keV, it is very difficult to perform accurate measurements of the required parameters and as a result there are very large discrepancies in the simulated data. With the current MC models, any predictions made in this energy range should be considered with caution.

More research is required to acquire accurate experimental data (i.e., cross-sections) for particle interactions in the low energy range (<500 keV for protons) so that more accurate semiempirical models can be developed.

## Figures and Tables

**Figure 2:**
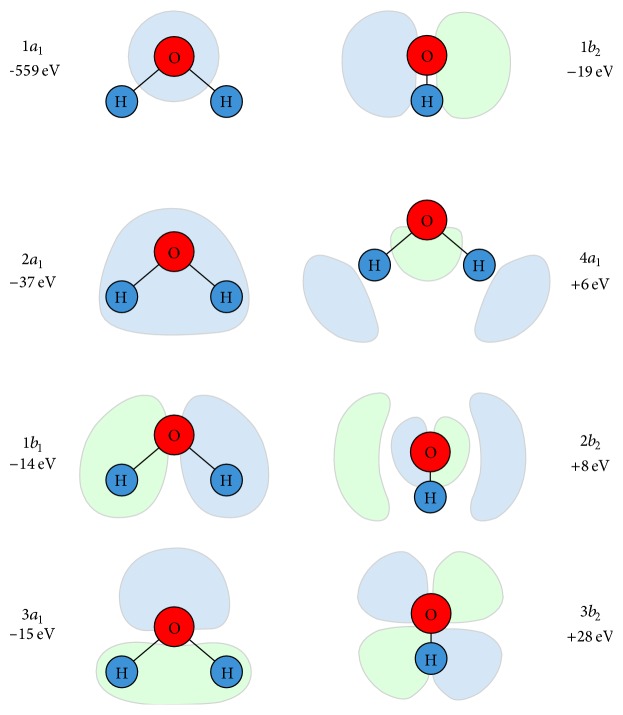
The five occupied (negative electron binding energy) and lowest three unoccupied (positive electron binding energy) molecular orbitals of an isolated water molecule. Calculated using the restricted Hartree-Fock wave function [34].

**Table 1:** Semiempirical parameters used for Rudd's ionisation model in [8].

Parameter	Liquid	Inner shells
*A*_1_	0.94	1.31
*A*_2_	1.13	0.37
*B*_1_	2.30	0.00
*B*_2_	22.0	0.00

**Table 2:** Semiempirical parameters used for Seltzer's ionisation model in [8]. *N*_*i*_ is the number of electrons per orbital, *B*_*i*_ is the ionisation energy of the orbital, *U*_*i*_ is the mean kinetic energy of the target electron in the orbital, and 〈*r*〉_*i*_ is the expected value of the radius of the *i*th orbital of the water molecule [8]. Orbital diagrams are shown in Figure 2.

*i*	Orbital	*N*_*i*_	*B*_*i*_ (eV)	*U*_*i*_ (eV)	〈*r*〉_*i*_ (Å)
1	^1^ *b*_1_	2	11.50	30	0.833
2	^3^ *a*_1_	2	11.75	40	0.867
3	^1^ *b*_2_	2	13.51	50	0.901
4	^2^ *a*_1_	2	16.0	60	0.906
5	^1^ *a*_1_	2	539.7	700	0.129

**Table 3:** Geant4-DNA physics models.

Process	Model	Energy range
Electron ionisation	Born ionisation model [13]	11 eV–1 MeV

Electronic excitation	Born excitation model [32, 33]	9 eV–1 MeV

Proton ionisation	Rudd ionisation model [22]	0 eV–500 keV
	Born ionisation model [32, 33]	500 keV–100 MeV

**Table 4:** RITRACKS physics models.

Process	Model	Energy range
Electron ionisation	Rudd ionisation model [5]	1 eV–50 keV
Electron ionisation	Seltzers ionisation model [6, 7]	>50 keV

Electronic excitation	Kaplan and Sukhonosov [10]	<50 eV–100 eV
Electronic excitation	Kutcher and Green [9]	>100 eV

Proton ionisation	Rudd ionisation model [22]	<10 MeV
